# Genetic association and causal relationship between multiple modifiable risk factors and autoimmune liver disease: a two-sample mendelian randomization study

**DOI:** 10.1186/s12967-024-05247-y

**Published:** 2024-05-04

**Authors:** Weize Gao, Chong Peng, Zhan Wang, Yongxin Li, Mingjun Liu

**Affiliations:** Department of Clinical Laboratory, Key Laboratory of Laboratory Medicine, The Affiliated Hospital of Qingdao University, Qingdao, 266003 China

**Keywords:** Autoimmune liver disease, Mendelian randomization, Modifiable risk factors, Autoimmune hepatitis, Primary biliary cholangitis, Primary sclerosing cholangitis

## Abstract

**Background:**

The intricate etiology of autoimmune liver disease (AILD) involves genetic, environmental, and other factors that yet to be completely elucidated. This study comprehensively assessed the causal association between genetically predicted modifiable risk factors and AILD by employing Mendelian randomization.

**Methods:**

Genetic variants associated with 29 exposure factors were obtained from genome-wide association studies (GWAS). Genetic association data with autoimmune hepatitis (AIH), primary biliary cholangitis (PBC) and primary sclerosing cholangitis (PSC) were also obtained from publicly available GWAS. Univariate and multivariate Mendelian randomization analyses were performed to identify potential risk factors for AILD.

**Results:**

Genetically predicted rheumatoid arthritis (RA) (OR = 1.620, 95%CI 1.423–1.843, *P* = 2.506 × 10^− 13^) was significantly associated with an increased risk of AIH. Genetically predicted smoking initiation (OR = 1.637, 95%CI 1.055–2.540, *P* = 0.028), lower coffee intake (OR = 0.359, 95%CI 0.131–0.985, *P* = 0.047), cholelithiasis (OR = 1.134, 95%CI 1.023–1.257, *P* = 0.017) and higher C-reactive protein (CRP) (OR = 1.397, 95%CI 1.094–1.784, *P* = 0.007) were suggestively associated with an increased risk of AIH. Genetically predicted inflammatory bowel disease (IBD) (OR = 1.212, 95%CI 1.127–1.303, *P* = 2.015 × 10^− 7^) and RA (OR = 1.417, 95%CI 1.193–1.683, *P* = 7.193 × 10^− 5^) were significantly associated with increased risk of PBC. Genetically predicted smoking initiation (OR = 1.167, 95%CI 1.005–1.355, *P* = 0.043), systemic lupus erythematosus (SLE) (OR = 1.086, 95%CI 1.017–1.160, *P* = 0.014) and higher CRP (OR = 1.199, 95%CI 1.019–1.410, *P* = 0.028) were suggestively associated with an increased risk of PBC. Higher vitamin D_3_ (OR = 0.741, 95%CI 0.560–0.980, *P* = 0.036) and calcium (OR = 0.834, 95%CI 0.699–0.995, *P* = 0.044) levels were suggestive protective factors for PBC. Genetically predicted smoking initiation (OR = 0.630, 95%CI 0.462–0.860, *P* = 0.004) was suggestively associated with a decreased risk of PSC. Genetically predicted IBD (OR = 1.252, 95%CI 1.164–1.346, *P* = 1.394 × 10^− 9^), RA (OR = 1.543, 95%CI 1.279–1.861, *P* = 5.728 × 10^− 6^) and lower glycosylated hemoglobin (HbA1c) (OR = 0.268, 95%CI 0.141–0.510, *P* = 6.172 × 10^− 5^) were positively associated with an increased risk of PSC.

**Conclusions:**

Evidence on the causal relationship between 29 genetically predicted modifiable risk factors and the risk of AIH, PBC, and PSC is provided by this study. These findings provide fresh perspectives on the management and prevention strategies for AILD.

**Supplementary Information:**

The online version contains supplementary material available at 10.1186/s12967-024-05247-y.

## Introduction

Autoimmune liver disease (AILD) is a collection of liver pathologies resulting from autoimmune dysregulation, distinguished by liver lymphocyte infiltration, heightened levels of circulating immunoglobulins, elevated liver enzyme activity, and the generation of autoantibodies [1]. The group of diseases under consideration can be categorized into three main types based on clinical presentation, biochemistry, imaging, and histopathology [1–3]. Autoimmune hepatitis (AIH) is defined by injury to the parenchymal cells of the liver, specifically interfacial hepatitis [4, 5]. Primary biliary cholangitis (PBC) is characterized by non-suppurative, destructive injury and cholestasis of the interlobular bile ducts [3, 6, 7]. Lastly, primary sclerosing cholangitis (PSC) is identified by the presence of multilayered onion-skin-like fibrosis in the intermediate-sized intra- and extra-hepatic bile ducts, along with multifocal bile duct obstruction [8, 9]. Contemporary incidence rates of disease per 100,000 range from 0.84 to 2.75 for PBC, 0.1 to 4.39 for PSC and 0.4 to 2.39 for AIH [1, 10, 11].

AILD is known to progress slowly with a malignant tendency, potentially culminating in conditions such as liver fibrosis, cirrhosis, and even hepatocellular carcinoma [11–13]. Unfortunately, there is currently no curative treatment for AILD, with corticosteroids in combination with azathioprine being the primary first-line medication for AIH [14]. Additionally, drugs such as ursodeoxycholic acid (UDCA), hormonal medications, and immunosuppressive agents show some therapeutic efficacy for patients with PSC and PBC, although a subset of patients may exhibit poor response. For those in end-stage AILD, the quality of life is severely compromised, often necessitating liver transplantation as a final recourse [3, 14–18].

Several previous studies have initially explored the association between certain modifiable risk factors and AILD risk. About one-third of AILD patients are accompanied by extrahepatic autoimmune diseases, including rheumatoid arthritis (RA), systemic lupus erythematosus (SLE), inflammatory bowel disease (IBD), and psoriasis [19–22]. Nonetheless, no comprehensive observational study has been conducted to elucidate the causal connection between the occurrence of these extrahepatic autoimmune disorders and AILD. In addition to being frequently associated with extrahepatic autoimmune diseases, osteoporosis is one of the most serious complications of PBC, leading to an increased risk of fracture [23–27]. Several factors affecting bone metabolism may be responsible for osteoporosis in patients with PBC, such as calcium dysregulation and vitamin D_3_ deficiency [25–27]. Previous studies have demonstrated that serum vitamin D_3_ levels in AIH and PBC patients are significantly lower than those in healthy controls, and are negatively correlated with liver fibrosis and cholestasis, respectively [4, 28–30]. Vitamin D_3_ deficiency may be a contributory factor in the development of AILD, but there are still few relevant studies [31–34]. AILD often presents a gender-dependent pattern. Peroxisome proliferator activated receptor alpha (PPAR-α) plays a crucial role in the innate immune defense [35]. Suppression of PPAR-α expression in female patients with PBC is accompanied by reduced testosterone levels, which should raise interest in the role of testosterone in the development of PBC [36–38]. In addition to the above factors, the association between many other modifiable risk factors and AILD has not been fully investigated, such as lifestyle, serum parameters, glucose metabolism, lipid metabolism, and obesity characteristics.

Mendelian randomization (MR) is a data analysis technique for assessing etiological inferences in epidemiological studies, based on the widely published genome-wide association study (GWAS) pooled datasets, which employs genetic variations as instrumental variables (IVs) to estimate the causal relationship between possible exposures and the outcome of interest [39, 40]. MR exploits the fact that genes are fixed and Mendel’s first and second laws of inheritance [41, 42]. It is a method comparable to randomized controlled trials without bias due to unobserved confounders, measurement errors and reverse causality [41, 43]. In the absence of randomized controlled studies, MR can be a time-saving and cost-effective way to assess and screen for potential causal associations, making it also known as " randomized controlled study built by nature”.

The unsatisfactory pharmacologic treatment of AILD underlines the imperative need to thoroughly investigate the etiology. Therefore, this study explores the causal effects of 29 genetically predicted modifiable risk factors on the risk of AIH, PBC, and PSC. This work aims to provide a comprehensive overview of the potential modifiable risk factors for AILD and offer new insights into the etiology of AILD.

## Methods

### MR design

**Fig. 1:**
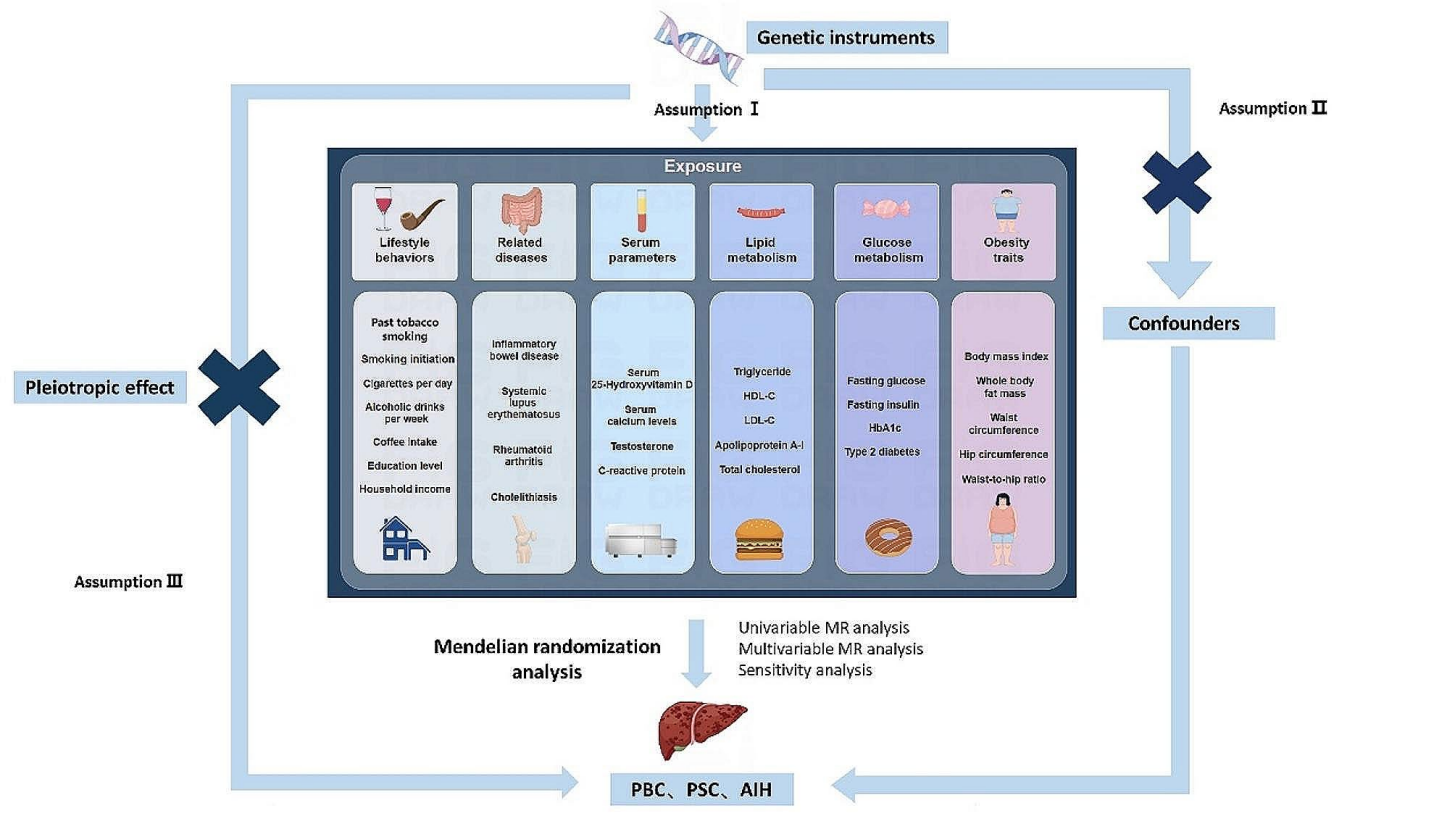
Visualization of methods and designs in this study. AIH, autoimmune hepatitis; PBC, primary biliary cholangitis; PSC, primary sclerosing cholangitis; HDL-C, high density lipoprotein cholesterol; LDL-C, low-density lipoprotein cholesterol; HbA1c, glycated hemoglobin

MR was used to explore causal associations between modifiable risk factors and different AILDs. MR is based on three key assumptions: (i) IVs are related to exposure; (ii) IVs are independent of confounders that may affect the exposure-outcome association; (iii) there is no way for IVs to affect the outcome other than by being related to exposure (Fig. 1). A total of 29 main risk factors were selected, which were divided into 6 categories: lifestyle behaviors, related diseases, serum parameters, lipid metabolism, glucose metabolism and obesity characteristics. Single nucleotide polymorphisms (SNPs) associated with these risk factors were extracted as IVs. The data sets used in this study were all sourced from public databases and received ethical approval prior to implementation. Therefore, no additional ethical approval was required for this study.

### IVs selection

Rigorous fltering steps were performed to control the IVs quality before MR analysis. The significant and independent SNPs for all factors were chosen as IVs based on the following criteria: [1] genome-wide association significance threshold of p-value < 5 × 10^− 8^; [2] all SNPs are independent with the threshold of LD r^2^ < 0.01 in a 10 Mb window. SNPs were also ruled out if palindromic sequences with intermediate allele frequencies were present. Proxy SNPs were utilized when merging exposure and outcome data [3]. The PhenoScanner was used to remove SNPs associated with confounders (http://www.phenoscanner.medschl.cam.ac.uk/*).* [4] Steiger filtering was applied to test the direction of causality of each SNP on exposure and outcome. SNPs meeting the criterion of “FALSE and *P* < 0.05” were excluded [5]. The MR-pleiotropy residual sum and outlier (MR-PRESSO) test was used to remove potential outlier SNPs [6]. For each SNP, the F statistic was determined by executing beta2 /se2. F > 10 is considered to have sufficient strength for the selected IVs. In this study, all F statistics meet F > 10. Detailed information on IVs for all exposure is provided in the supplementary material.

### GWAS summary of autoimmune liver disease and baseline characteristics of 29 candidate factors

IVs associated with AIH were obtained from the largest atlas of genetic associations for 220 human phenotypes to date, including 484,413 European controls and 821 European patients (GWAS ID: ebi-a-GCST90018785). The IVs associated with PBC were extracted from the largest international genome-wide meta-analysis of PBC to date, which included 16,489 European controls and 8021 European patients (GWAS ID: ebi-a-GCST90061440). For PSC, the IVs were obtained from the most extensive PSC GWAS conducted by the International PSC Study Group (IPSCSG), including 12,019 European controls and 2871 European patients (GWAS ID: ieu-a-1112). Twenty-nine potential risk factors were included in the analysis. Risk factors can be divided into six categories: lifestyle, related diseases, serum parameters, lipid metabolism, glucose metabolism, and obesity characteristics (Table 1). Lifestyle behaviors include smoking, alcohol consumption, coffee consumption, educational attainment, and household income. Related diseases include IBD, RA, SLE and cholelithiasis. Serum parameters include serum 25-Hydroxyvitamin D (vitamin D_3_), calcium, C-reactive protein (CRP), and testosterone. In addition, five traits related to lipid metabolism, four traits related to glucose metabolism and five traits related to obesity were analyzed. For each of the 29 modifiable potential risk factors examined, the F statistic of their respective genetic tools was greater than the empirical threshold of 10, indicating no potential weak tool bias. Ethical approval was obtained for all selected GWAS and informed consent was obtained from individuals.

**Table 1:**
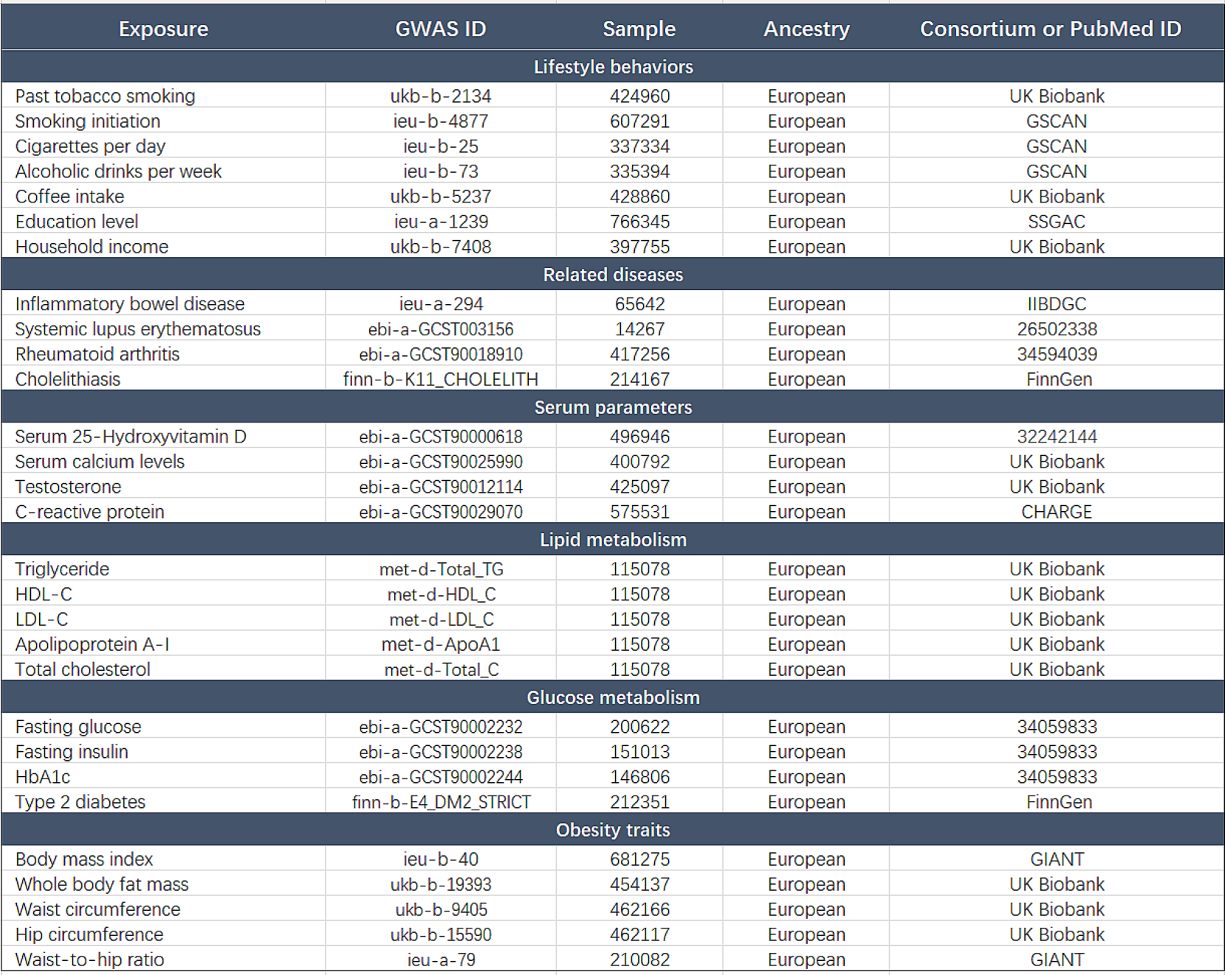
Baseline characteristics of 29 candidate factors. GSCAN, gwas and sequencing consortium of alcohol and nicotine use; SSGAC, social science genetic association consortium; IIBDGC, international inflammatory bowel disease genetics consortium; CHARGE, the cohorts for heart and aging research in genomic epidemiology consortium; GIANT, genetic investigation of anthropological traits consortium

### Genetic correlation analysis

Linkage disequilibrium score (LDSC) regression was performed to determine the genetic correlation of 29 potential risk factors with AIH, PBC and PSC, as well as to evaluate the extent of sample overlap [44]. The regression intercept of bivariate LDSC reflected the sample overlap of trait pairs. GWAS summary statistics were filtered according to HapMap3 ref. Variants that were not SNPs (e.g., indels) and SNPs that were strand-ambiguous, repeated, and had a minor allele frequency (MAF) < 0.01 were excluded. The LDSC examines the association between test statistics and linkage disequilibrium to quantify the contribution of inflation from a true polygenic signal or bias [45]. This method can evaluate genetic correlation from GWAS summary statistics and is not biased by sample overlap [44]. The z-scores of each variant from Trait 1 are multiplied by the z-scores of each variant from Trait 2. The genetic covariance was estimated by regressing this product against the LD score. The genetic covariance normalized by SNP-heritability represents the genetic correlation. A Bonferroni-corrected *P*-value was set as 0.05/29 (1.72 × 10^− 3^). *P* < 1.72 × 10^− 3^ was defined as statistically significant association.

### Univariable MR analyses

Univariate MR analyses used the random-effect inverse variance weighted (IVW) method as the primary analysis to estimate the association between modifiable risk factors and the risk of PBC, PSC, and AIH. In addition, MR-Egger and weighted median were utilized to refine the IVW estimates as they provide more robust estimates in a wider range of scenarios despite being less efficient (wider CIs). If the effect estimation in the IVW technique was significant and no contradictory outcomes were obtained in other methods, the causal connection was considered suggestive. In this study, multiple techniques for sensitivity analysis were implemented. First, the heterogeneity of IVs was evaluated using Cochran’s Q test. Specifically, heterogeneity was detected if the P value of the Cochran Q test was less than 0.05. Furthermore, the MR-Egger regression intercept, MR-PRESSO (global test), leave-one-out analysis, funnel plot and forest plot were employed to check for any potential pleiotropy and assessed the robustness of the results. When the p-value was less than 0.05, the MR analysis might support the premise that IVs had a direct effect on the outcome (conflicts with MR hypothesis III). Besides, leave-one-out analysis was performed to determine whether the causal estimate was driven by any single SNP. The power values were calculated online to enhance the robustness of the findings (https://shiny.cnsgenomics.com/mRnd/*).* All findings of MR analysis, sensitivity analysis, and visualization plots are available in the Supplementary Material.

### Multivariable MR analyses

Multivariate MR was performed only for the phenotypes of interest (lipid metabolism, glucose metabolism, obesity characteristics). A Bonferroni-corrected *P*-value was set as 0.05/29 (1.72 × 10^− 3^). *P* values ranging from 1.72 × 10^− 3^ to 0.05 were classified as suggestive causal associations. Results are reported as ORs and corresponding 95% confidence intervals (CIs). All the analyses were undertaken using R 4.3.1 (R Foundation for statistical Computing, Vienna, Austria).

## Results

### LDSC regression analysis

MR estimates may violate causality provided there is a genetic correlation between exposure and outcome. In this study, no significant genetic correlation was observed between 29 potential risk factors and AIH, PBC and PSC. This suggested that MR estimates were not confounded by shared genetic components. Furthermore, the regression intercepts of the bivariate LDSC regressiom were not significantly deviated from zero. This reflected the extremely limited sample overlap between all exposures and outcomes. Detailed information regarding all genetic correlation results is available in the Supplementary Materials.

### Causal effects of the modifiable risk factors on AIH

**Fig. 2:**
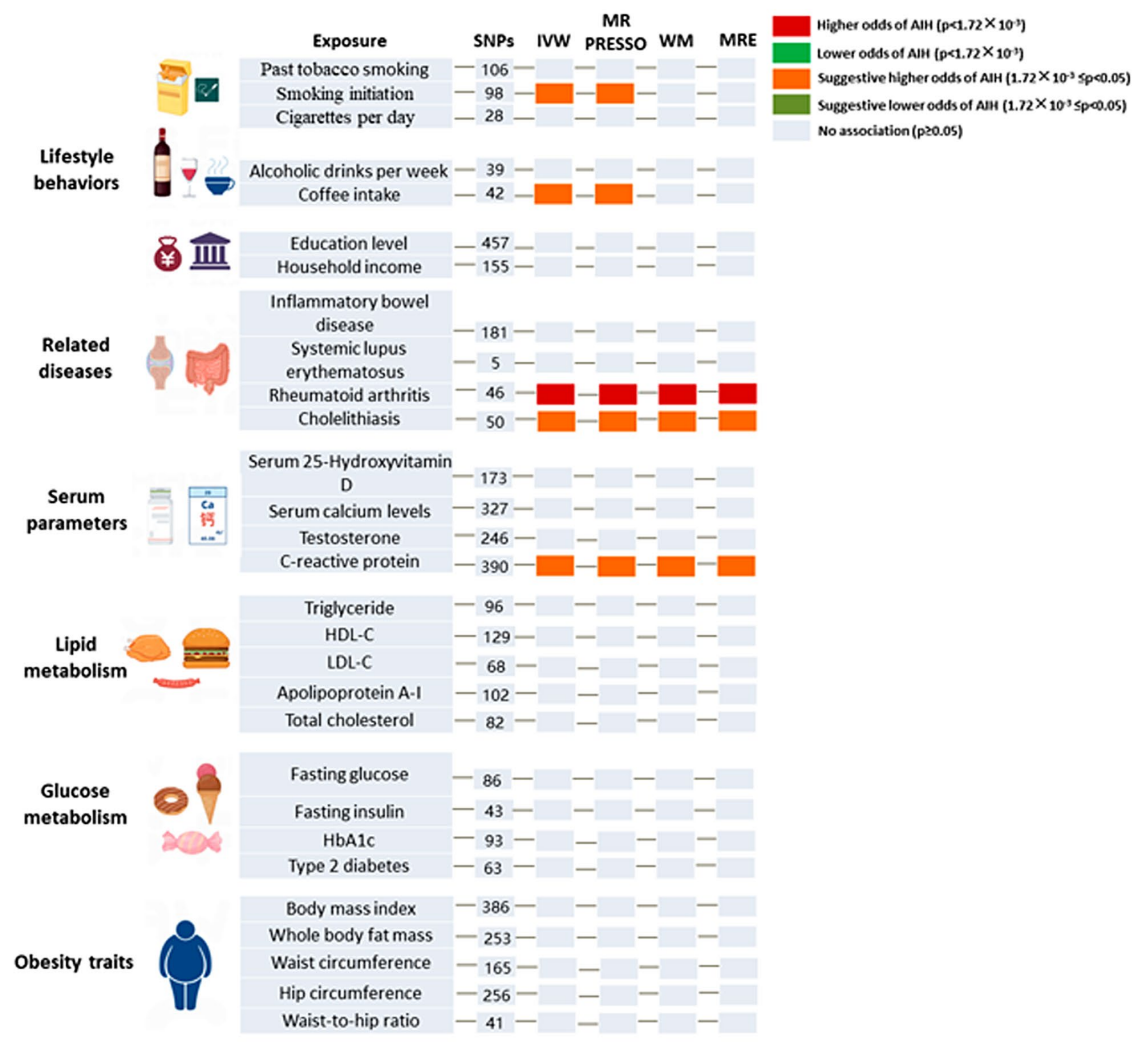
The causal effect of modifiable risk factors on AIH. AIH, autoimmune hepatitis; HDL-C, high density lipoprotein cholesterol; LDL-C, low-density lipoprotein cholesterol; HbA1c, glycated hemoglobin

Univariate MR Analysis showed that in the lifestyle segment, genetically predicted smoking initiation (OR = 1.637, 95%CI 1.055–2.540, *P* = 0.028) and lower coffee intake (OR = 0.359, 95%CI 0.131–0.985, *P* = 0.047) were suggestively associated with an increased risk of AIH (Fig. 2). No pleiotropy (MR-Egger regression intercept: *P* = 0.483; MR-PRESSO-Global Test: *P* = 0.198) or heterogeneity (Cochran’s Q test: *P* = 0.212) was detected for smoking initiation. No pleiotropy (MR-Egger regression intercept: *P* = 0.724; MR-PRESSO-Global Test: *P* = 0.712) or heterogeneity (Cochran’s Q test: *P* = 0.679) was detected for coffee intake. In the related disease plate, the genetically predicted extrahepatic autoimmune disease RA (OR = 1.620, 95%CI 1.423–1.843, *P* = 2.506 × 10^− 13^) was significantly associated with an increased risk of AIH. No pleiotropy (MR-Egger regression intercept: *P* = 0.304; MR-PRESSO-Global Test: *P* = 0.081) or heterogeneity (Cochran’s Q test: *P* = 0.074) was detected for RA. Genetically predicted cholelithiasis had a suggestive increased risk for AIH (OR = 1.134, 95%CI 1.023–1.257, *P* = 0.017). No pleiotropy (MR-Egger regression intercept: *P* = 0.312; MR-PRESSO-Global Test: *P* = 0.767) or heterogeneity (Cochran’s Q test: *P* = 0.783) was detected for cholelithiasis. In serum parameters profile, higher CRP (OR = 1.397, 95%CI 1.094–1.784, *P* = 0.007) was a suggestive adverse factor for AIH. Heterogeneity (Cochran’s Q test: *P* = 0.066) or pleiotropy (MR-Egger regression intercept: *P* = 0.269; MR-PRESSO-Global Test: *P* = 0.052) was not found in CRP. For the positive findings above, no outliers were identified with MR-PRESSSO and the leave-one-out plot as well as funnel plots. All findings of MR analysis, sensitivity analysis, and visualization plots are available in the Supplementary Materials.

### Causal effects of the modifiable risk factors on PBC

**Fig. 3:**
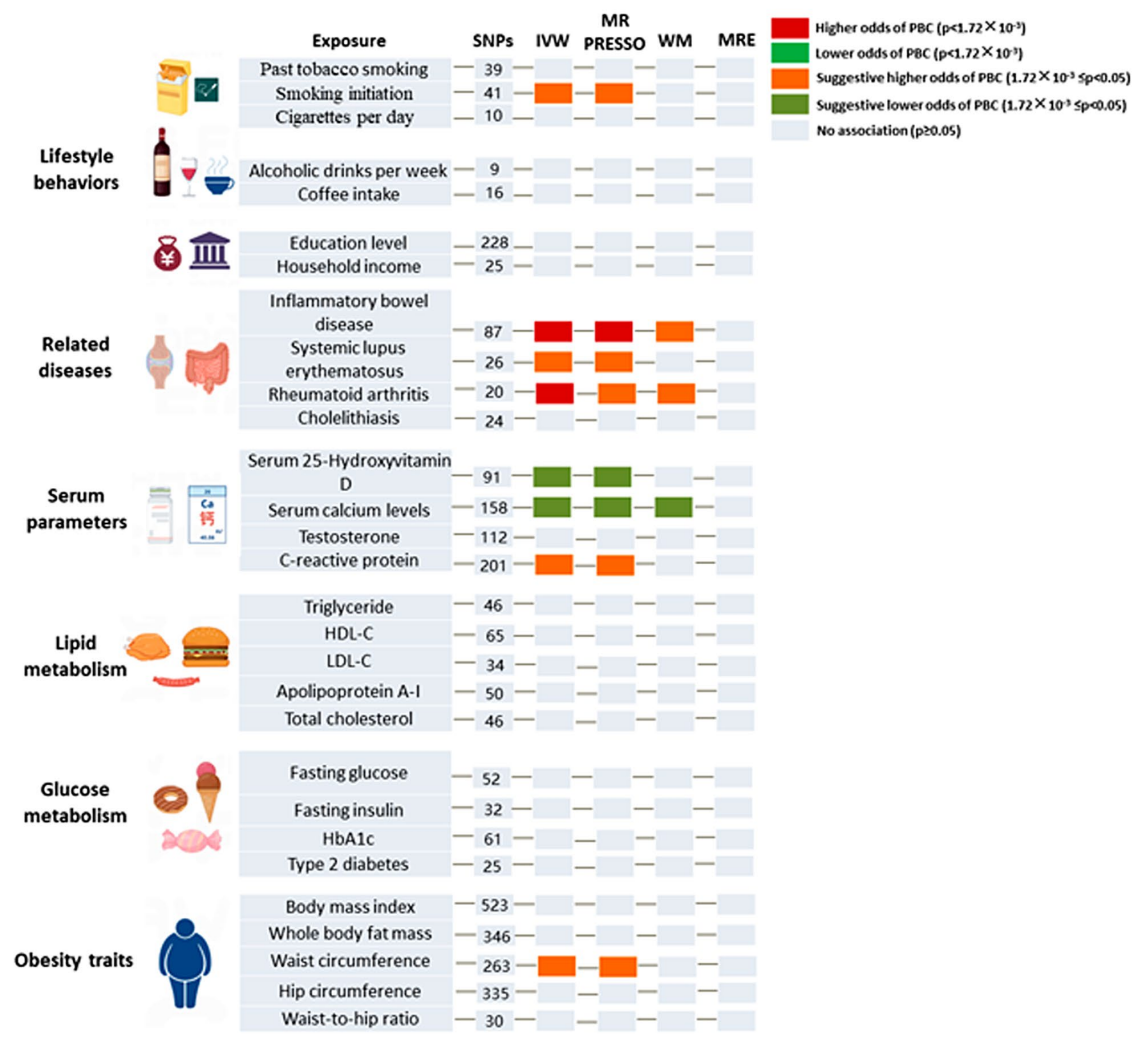
Causal effects of the modifiable risk factors on PBC. PBC, primary biliary cholangitis; HDL-C, high density lipoprotein cholesterol; LDL-C, low-density lipoprotein cholesterol; HbA1c, glycated hemoglobin

Univariate analysis showed that genetically predicted smoking initiation (OR = 1.167, 95%CI 1.005–1.355, *P* = 0.043) was suggestively associated with an increased risk of PBC (Fig. 3). No pleiotropy (MR-Egger regression intercept: *P* = 0.164; MR-PRESSO-Global Test: *P* = 0.998) or heterogeneity (Cochran’s Q test: *P* = 0.999) was detected for smoking initiation. In the related disease plate, genetically predicted extrahepatic autoimmune diseases IBD (OR = 1.212, 95%CI 1.127–1.303, *P* = 2.015 × 10^− 7^) and RA (OR = 1.417, 95%CI 1.193–1.683, *P* = 7.193 × 10^− 5^) were significantly associated with increased risk of PBC. Heterogeneity was observed with a Cochran Q-derived *P* value < 0.05 for IBD and RA. As the random-effects IVW was used as main result, heterogeneity is acceptable. No pleiotropy was detected for IBD (MR-Egger regression intercept: *P* = 0.227; MR-PRESSO-Global Test: *P* = 0.097) and RA (MR-Egger regression intercept: *P* = 0.874; MR-PRESSO-Global Test: *P* = 0.117). Besides, genetically predicted SLE had a suggestive increased risk for PBC (OR = 1.086, 95%CI 1.017–1.160, *P* = 0.014). No pleiotropy was detected for SLE (MR-Egger regression intercept: *P* = 0.358). Heterogeneity was observed for SLE with a Cochran Q-derived *P* value < 0.05, but acceptable. In serum parameters profile, higher vitamin D_3_ (OR = 0.741, 95%CI 0.560–0.980, *P* = 0.036) and calcium (OR = 0.834, 95%CI 0.699–0.995, *P* = 0.044) levels were suggestive protective factors for PBC with possible heterogeneity (Cochran’s Q test: *P* = 2.140 × 10^− 3^ ;*P* = 2.244 × 10^− 3^, respectively). But no pleiotropy was detected for serum vitamin D_3_ (MR-Egger regression intercept: *P* = 0.813) and calcium (MR-Egger regression intercept: *P* = 0.419) levels. However, genetically predicted higher CRP had a suggestive increased risk for PBC (OR = 1.199, 95%CI 1.019–1.410, *P* = 0.028). Heterogeneity was observed for CRP (Cochran’s Q test: *P* = 4.545 × 10^− 4^), but no pleiotropy was detected (MR-Egger regression intercept: *P* = 0.747). Among obesity characteristics, higher waist circumference was suggestively associated with an increased risk of PBC (OR = 1.272, 95%CI 1.033–1.567, *P* = 0.024). No pleiotropy (MR-Egger regression intercept: *P* = 0.921; MR-PRESSO-Global Test: *P* = 0.053) but slight heterogeneity (Cochran’s Q test: *P* = 0.048) was detected for waist circumference. All findings of MR analysis, sensitivity analysis, and visualization plots are available in the Supplementary Materials.

### Causal effects of the modifiable risk factors on PSC

**Fig. 4:**
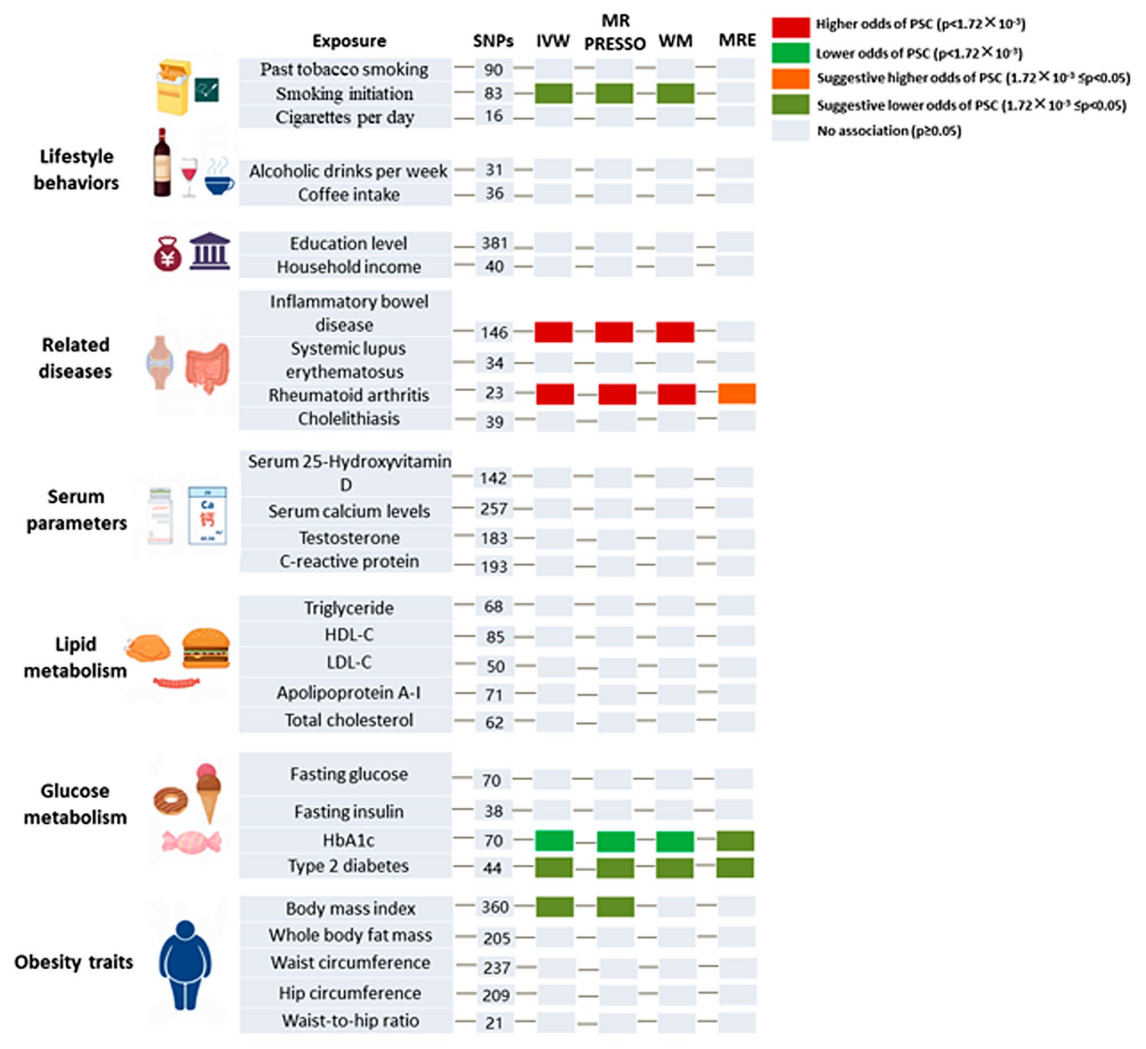
Causal effects of the modifiable risk factors on PSC. PSC, primary sclerosing cholangitis; HDL-C, high density lipoprotein cholesterol; LDL-C, low-density lipoprotein cholesterol; HbA1c, glycated hemoglobin

Univariate MR Analysis showed that genetically predicted smoking initiation (OR = 0.630, 95%CI 0.462–0.860, *P* = 0.004) was suggestively associated with a decreased risk of PSC in the lifestyle segment (Fig. 4). No pleiotropy (MR-Egger regression intercept: *P* = 0.670; MR-PRESSO-Global Test: *P* = 0.196) or heterogeneity (Cochran’s Q test: *P* = 0.183) was detected for smoking initiation. In addition, genetically predicted IBD (OR = 1.252, 95%CI 1.164–1.346, *P* = 1.394 × 10^− 9^) and RA (OR = 1.543, 95%CI 1.279–1.861, *P* = 5.728 × 10^− 6^) were positively associated with an increased risk of PSC. No pleiotropy (MR-Egger regression intercept: *P* = 0.349) but heterogeneity (Cochran’s Q test: *P* = 8.392 × 10^− 4^) was detected for IBD. No pleiotropy (MR-Egger regression intercept: *P* = 0.635; MR-PRESSO-Global Test: *P* = 0.161) or heterogeneity (Cochran’s Q test: *P* = 0.149) was detected for RA. In the glucometabolic plate, genetically predicted glycosylated hemoglobin (HbA1c) (OR = 0.268, 95%CI 0.141–0.510, *P* = 6.172 × 10^− 5^) was a protective factor for PSC. No pleiotropy (MR-Egger regression intercept: *P* = 0.721; MR-PRESSO-Global Test: *P* = 0.190) or heterogeneity (Cochran’s Q test: *P* = 0.171) was detected for HbA1c. Besides, genetically predicted type 2 diabetes had a suggestive decreased risk for PSC (OR = 0.877, 95%CI 0.780–0.986, *P* = 0.028). No pleiotropy (MR-Egger regression intercept: *P* = 0.145; MR-PRESSO-Global Test: *P* = 0.242) or heterogeneity (Cochran’s Q test: *P* = 0.327) was detected for type 2 diabetes. Among obesity characteristics, higher BMI was suggestively associated with a decreased risk of PSC (OR = 0.777, 95%CI 0.626–0.964, *P* = 0.022). No pleiotropy (MR-Egger regression intercept: *P* = 0.585; MR-PRESSO-Global Test: *P* = 0.090) or heterogeneity (Cochran’s Q test: *P* = 0.104) was detected for BMI. All findings of MR analysis, sensitivity analysis, and visualization plots are available in the Supplementary Materials.

### Multivariable MR analysis of AIH, PBC and PSC

Only the interrelated genetically predicted modifiable risk factors were adjusted for each other in the multivariate MR analysis model. Obesity features, glucose metabolism, and lipid metabolism are the key hereditary predicted modifiable risk factors that need to be adjusted. Consistent with the findings of univariate analysis, genetically predicted levels of TG (OR = 0.884, 95%CI 0.646–1.210, *P* = 0.442), HDL(OR = 0.919, 95%CI 0.685–1.233, *P* = 0.575), LDL (OR = 0.897, 95%CI 0.655–1.229, *P* = 0.500), and ApoA1 (OR = 0.870, 95%CI 0.656–1.154, *P* = 0.334) were not significantly causally associated with the risk of AIH after adjusting for genetically predicted HDL/LDL, TG/LDL, TG/HDL, and TG/LDL, respectively. Fasting glucose (OR = 1.230, 95%CI 0.593–2.550, *P* = 0.577), fasting insulin (OR = 2.101, 95%CI 0.731–6.036, *P* = 0.168), and HbA1c levels (OR = 0.483, 95%CI 0.186–1.253, *P* = 0.135), as well as genetically predicted type 2 diabetes (OR = 0.926, 95%CI 0.777–1.103, *P* = 0.388) were also not associated with the risk of AIH after correction for each other. In multivariate analysis, obesity parameters were reconfirmed to be independent from the risk of AIH.

Similar to the multivariate analysis of AIH, multivariate adjustment did not modify the conclusion that indicators of lipid and glucose metabolism had no association with PBC risk. Notably, higher waist circumference (OR = 1.578, 95%CI 0.236–4.537, *P* = 0.638), which was suggestively linked with an increased risk of PBC in univariate analysis, was no longer suggestively associated with PBC after adjusting for other obesity characteristics in the multivariate analysis.

Finally, in the multivariate analysis of PSC, after correction for genetically fasting glucose/fasting insulin/type 2 diabetes, the suggestive association of HbA1c (OR = 0.369, 95%CI 0.138–0.986, *P* = 0.046) with a decreased risk of PSC remained significant. However, higher BMI (OR = 0.763, 95%CI 0.344–1.691, *P* = 0.505), which was suggestively linked with an increased risk of PSC in univariate analysis, was no longer associated with PSC after adjusting for other obesity characteristics in the multivariate analysis.

## Discussion

Significant changes have been reported in the epidemiology of AILD [1, 10]. AIH and PSC incidence and prevalence in Europe are on the rise. The prevalence of PBC is also increasing in Europe, North America and Asia Pacific [1, 5, 10]. Overall, although AILD is rare, its clinical burden is disproportionately high with regard to population incidence and prevalence [1]. Age, gender, and race also affect clinical outcomes [38]. Patient morbidity and mortality are mirrored in the high demand for gastroenterology, hepatology and organ transplantation services [46]. In this study, causal associations of 29 potential risk factors with AIH, PBC, and PSC were systematically explored by MR.

Smoking is associated with numerous autoimmune diseases, leading to diverse effects such as tissue damage, apoptosis, inflammation, and anti-estrogen effects [47]. Several previous large epidemiological studies have revealed a strong association between smoking, or a history of smoking, and the risk of PBC [48–51]. Notably, a history of smoking is particularly linked to the presence of advanced fibrosis in PBC patients [47, 52–54]. As the number of cigarettes smoked (pack-years) increased, so did the risk of developing advanced fibrosis in PBC patients [54]. Comparisons have shown a significant difference in liver inflammatory activity between smokers and non-smokers, with smokers exhibiting elevated levels of IL-10 and IFN-γ, reflective of a Th1 response [55]. Furthermore, hydrocarbons contained in cigarette smoke have been found to be associated with PBC population colonies [56]. The MR Analysis results in this study further confirmed that smoking is a suggestive risk factor for PBC. However, the link between smoking and AIH risk is still debated [57]. This study showed that genetically predicted smoking was suggestively associated with an increased risk of AIH. While further investigation is required to fully characterize and consolidate the aforementioned results, it is discernible that individuals with PBC and AIH have to be discouraged from smoking. Surprisingly, despite being under the same umbrella of AILD, the MR analysis results in the present study suggested that smoking was a potential protective factor for PSC patients. Moreover, previous retrospective studies have corroborated our findings. A UK cohort study by WEBB G J et al. found that non-smoking was linked to a lower prevalence of PSC [10]. Similarly, a case-control study by BOONSTRA K et al. indicated that smoking was associated with a reduced risk of developing PSC [58]. In the lifestyle panel, genetically predicted lower coffee intake was suggestively associated with an increased risk of AIH. This result aligns with previous studies indicating that individuals with AIH have lower lifetime coffee consumption than healthy controls [59].

In previous studies, several extra-hepatic autoimmune diseases, including SLE and RA, have been reported to be closely associated with AILD [60]. Notably, although AILD like AIH and PBC are considered rare, their co-existence with SLE in patients presenting with liver enzyme abnormalities is relatively common [61]. The overlap rates of SLE with AIH and PBC were 1.6-15% and 2.2-7.5%, respectively [62, 63]. Current MR results support a genetically predicted causal relationship between SLE and PBC. However, current findings suggest that genetically predicted SLE does not significantly alter the risk of AIH.

The most common co-existing AILD in RA is PBC, with a prevalence of 3.8–6.3% [64, 65]. While the incidence of RA in PBC is reported to be 1.8–13%, about 50% of patients with PBC show RF positive [66–68]. Genetic investigations have revealed shared genes between RA and PBC such as HLA-DQB1, STAT4, IRF5, MMEL1, and CTLA4 [69]. In addition, in patients with AIH, the prevalence of RA ranges from 1.6–5.4% [70]. Furthermore, a proteome-wide MR has highlighted AIF1 and HLA-DQA2 as targets for PSC and RA [71]. This study suggests a causal connection between genetically predicted RA and the susceptibility to PBC, AIH, and PSC. Patients with overlapping diseases are typically diagnosed with RA before AILD, underscoring the importance of screening for AMA and ANA in RA patients presenting with cholestatic elevated liver enzymes, as the co-occurrence of these conditions can impact the prognosis and management of patients.

Both ulcerative colitis (UC) and Crohn disease (CD) are associated with a variety of hepatobiliary symptoms. The majority of patients with PSC are initially diagnosed with extensive colitis [72]. Currently, there is an ongoing discussion regarding whether PSC represents an extraintestinal manifestation of IBD or if PSC and IBD are distinct entities that share a common susceptibility leading to a dual phenotype [19, 73]. This study reinforces the causative link between genetically predicted IBD and PSC. Given that PSC-IBD is associated with a higher risk of malignancy [74], timely identification of high-risk individuals and the implementation of appropriate surveillance strategies are crucial in managing this complex relationship. Furthermore, the findings of this study support a causative relationship between genetically predicted IBD and PBC risk, aligning with earlier studies conducted by Zhang and Zhao et al [75, 76]. Disruption of intestinal permeability in IBD may lead to bacterial translocation, bile duct cell activation, and liver inflammation, ultimately contributing to the onset of PBC [77]. To further elucidate the causal interplay between IBD and PBC, it is imperative to conduct long-term prospective studies.

The serum indicators analyzed in this MR Study highlighted Vitamin D_3_ as a potential protective factor against PBC, indicating a reduced risk associated with this nutrient. In patients with PBC, Vitamin D_3_ deficiency is a common occurrence. Several studies have identified its correlation with poor response to UDCA, increased risk of cirrhosis development, liver-related mortality, and the necessity for liver transplantation [28, 32, 34]. Therefore, these findings imply that Vitamin D_3_ supplementation could serve as a cost-effective strategy for early intervention in the management of PBC [38].

Regarding data sources and study design, this study presents several advantages. First, the study avoided the bias inherent in conventional observational epidemiological studies by utilizing univariate and multivariate multimodal MR analyzes to evaluate the causal link between two complicated genetic variables. Second, this study is the first to systematically analyze multiple modifiable causal risk factors for AILD.

The study still has a few shortcomings. Firstly, in this study, there are IVW results whose power value failed to exceed 80%, potentially impacting the credibility of the results. Furthermore, the Causal Analysis Using Summary Effect estimates (CAUSE) method should be employed to confirm whether the detected horizontal pleiotropy is relevant or irrelevant. CAUSE can avoid more false positives induced by correlated horizontal pleiotropy than other methods [78].

## Conclusion

Evidence on the causal relationship between 29 genetically predicted modifiable risk factors and the risk of PSC, PBC, and AIH is provided by this study. These findings provide fresh perspectives on the management and prevention strategies for AILD.

### Electronic supplementary material

Below is the link to the electronic supplementary material.

Supplementary Material 1

Supplementary Material 2

Supplementary Material 3

Supplementary Material 4

## Data Availability

The datasets used and analyzed during the current study are available from the corresponding author on reasonable request.
